# On the Effect of the Patient Table on Attenuation in Myocardial Perfusion Imaging SPECT

**DOI:** 10.1186/s40658-024-00713-4

**Published:** 2025-01-20

**Authors:** Tamino Huxohl, Gopesh Patel, Wolfgang Burchert

**Affiliations:** Institute of Radiology, Nuclear Medicine and Molecular Imaging, Heart and Diabetes Center North Rhine-Westphalia, University Hospital (Ruhr University Bochum), Medical Faculty OWL (Bielefeld University), Bad Oeynhausen, Germany

**Keywords:** SPECT, Myocardial perfusion imaging (MPI), Attenuation correction, Patient table

## Abstract

**Background:**

The topic of the effect of the patient table on attenuation in myocardial perfusion imaging (MPI) SPECT is gaining new relevance due to deep learning methods. Existing studies on this effect are old, rare and only consider phantom measurements, not patient studies. This study investigates the effect of the patient table on attenuation based on the difference between reconstructions of phantom scans and polar maps of patient studies.

**Methods:**

Jaszczak phantom scans are acquired according to quality control and MPI procedures. An algorithm is developed to automatically remove the patient table from the CT for attenuation correction. The scans are then reconstructed with attenuation correction either with or without the patient table in the CT. The reconstructions are compared qualitatively and on the basis of their percentage difference. In addition, a small retrospective cohort of 15 patients is examined by comparing the resulting polar maps. Polar maps are compared qualitatively and based on the segment perfusion scores.

**Results:**

The phantom reconstructions look qualitatively similar in both the quality control and MPI procedures. The percentage difference is highest in the lower part of the phantom, but it always remains below 17.5%. Polar maps from patient studies also look qualitatively similar. Furthermore, the segment scores are not significantly different (*p*=0.83).

**Conclusions:**

The effect of the patient table on attenuation in MPI SPECT is negligible.

## Background

Myocardial perfusion imaging (MPI) single-photon emission computed tomography (SPECT) is a widely used examination for the assessment of coronary artery disease (CAD). Attenuation caused by the body of the patient and the patient table reduces the interpretability of the examinations by decreasing the specificity [1–3]. To address this issue, non-diagnostic CT scans are routinely acquired using SPECT/CT hybrid scanners to perform attenuation correction. However, these CT scans have their own drawbacks, such as misregistration due to patient motion [4, 5] and additional radiation exposure. In addition, most SPECT devices are not SPECT/CT hybrid scanners [6, 7], so they lack attenuation correction capabilities. Consequently, there is a movement towards deep-learning attenuation correction (DLAC) [8–13].

Indirect DLAC involves training an artificial neural network using SPECT/CT data to predict an attenuation map from an uncorrected SPECT scan. While studies have demonstrated its efficacy, a significant issue remains in dealing with attenuation caused by the patient table. For example, in some cases, the patient table is not predicted by the DLAC model [8, 13]. These works argue that they do not consider the patient table because it is invisible in the SPECT scan, as it does not emit any radiation. In contrast, the patient table is predicted by the DLAC model in [9]. Unfortunately, this is not discussed in the article, but only known from the included images of the model predictions.

In this paper, the effect of the patient table on attenuation in MPI SPECT is investigated. In doing so, it helps to answer the question of whether DLAC models should aim to include the patient table in their predictions. While it seems that a DLAC model is able to predict the patient table well [9], it may hinder its transfer to other scanners, because it will likely always reproduce the patient table from its training data. In this work, the effect of the patient table on attenuation is measured in phantom scans and in polar maps of patient studies. If the effect is small, it is reasonable to train DLAC models that do not predict the patient table to allow easier transfer to other scanners with different patient tables.

Few studies have investigated the effect of the patient table on attenuation, since the CT corrects for patient table attenuation anyway. Thus, previous work has focused more on misregistration problems caused by the patient table [14, 15]. However, with respect to DLAC, the effect of the patient table on attenuation gains new relevance. There is a single, thirty-year-old paper that investigated the effect of different patient tables on attenuation in MPI SPECT [16]. O’Connor and Bothun evaluated how much the counts in a phantom scan were reduced when looking through the patient table at different angles. They found that the patient table reduced counts the most at angles of $90^\circ$ and $270^\circ$, where the radiation travels the longest distance through the patient table. Overall, most patient tables have a small effect, reducing counts by 2% to 12%.

Since this study is old and the issue is gaining new relevance, this work takes a new look at the effect of the patient table on attenuation. In contrast to [16], this work does not consider the reduction of counts in the projection images, but the difference in the reconstructed images. For this purpose, not only phantom scans are considered, but also data from a few patients. To investigate the effect of the patient table from a clinical perspective, polar maps from reconstructions with and without patient table attenuation are compared.

## Materials and methods

All scans examined in this paper were obtained with a Symbia Intevo SPECT/CT scanner (Siemens Healthineers, Erlangen, Germany). The scanner acquires SPECT projection images with two detector heads. Counts are accumulated for a photopeak energy of 129 keV to 150 keV. In addition, counts for a scatter energy window of 108 keV to 129 keV are used for scatter correction with the triple energy window method [17]. All images are reconstructed using ordered subset expectation maximization (OSEM) as implemented in the software of the manufacturer with 10 iterations and 3 subsets. Reconstructions have a resolution of $128\times 128\times 128$ voxels with a voxel size of $4.8\times 4.8\times 4.8$ $\hbox {mm}^3$. CT images are acquired with a peak voltage of 130 kV, a B08s convolutional kernel, and a variable number of slices. They have a resolution of $128\times 128\times \, X$ voxels (where *X* is the number of slices) with a voxel size of $1.0\times 1.0\times 5.0$ $\hbox {mm}^3$. They were manually registered to the SPECT images. Conversion of the CT images into attenuation maps and reconstruction with attenuation correction are performed using the software of the manufacturer.

To obtain attenuation-corrected reconstructions without the patient table, a custom algorithm^1^ was implemented to automatically remove the patient table from a CT scan. The algorithm works as follows: First, the central CT slice is selected and a threshold of -700 HU is applied. This creates a binary image with two regions: the upper region is the phantom or patient body and the lower region is the patient table. However, the patient table region is slightly smaller than the actual patient table. Thus, this region is slightly enlarged by applying three iterations of dilations with a $9\times 9$ kernel. The resulting region is removed from all CT slices. For each CT, the success of the automatic removal was visually verified.

To get a general idea of the influence of the patient table on attenuation, a scan of a Jaszczak phantom was acquired according to our quality control procedure. This means that 64 projections were acquired over a $360^\circ$ circular orbit with a parallel collimator. Subsequently, another scan of the Jaszczak phantom was acquired according to our MPI protocol. This means that 34 projections were acquired over a $204^\circ$ cardiocentric orbit using a smartzoom collimator [18]. The smartzoom collimator is a special collimator for MPI that focuses in the center and becomes nearly parallel at the edges. Phantom scans and the percentage difference between attenuation with and without the patient table are compared visually.

**Table 1:** Gender, age, height, weight and BMI of the patients and the imaging protocol in the data. M indicates male, F female, S stress and R rest. For the numerical values, the mean, the standard deviation, and the value range are given

Gender	Age (year)	Height (cm)	Weight (kg)	BMI	Protocol
10 M, 5 F	${71.3}\pm {9.2}$	${172.0}\pm {7.6}$	${88.1}\pm {19.1}$	${29.7}\pm {6.0}$	13 S, 6 R
	50 to 84	157 to 185	50 to 121	17.3 to 40.8	

In addition to the phantom scans, retrospective data was collected from 19 studies (rest and stress) of 15 patients. The clinical characteristics of the patients are shown in Table 1. The patient studies were processed similarly to the phantom studies. Polar maps according to the American Heart Association (AHA) model [19] were generated for each study using the Cedars-Sinai Cardiac Suite [20]. Segment perfusion scores were determined to be normally distributed using the Shapiro-Wilk test and, if so, differences were analysed using the t-test. Segment perfusion scores were compared globally and per segment.

## Results

**Fig. 1:**
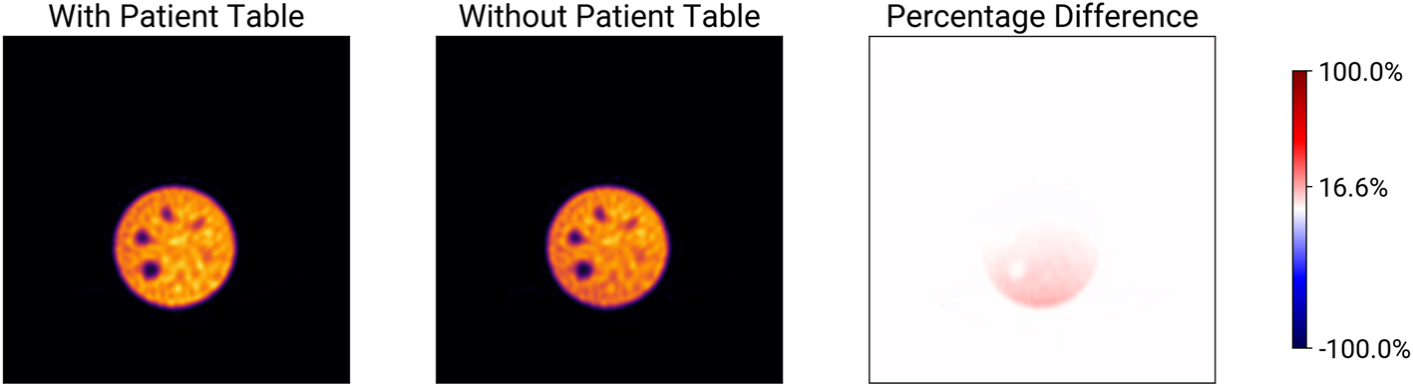
Difference of reconstructions of a Jaszczak phantom acquired with a parallel collimator over a $360^\circ$ radius according to our quality control procedure. From left to right: Reconstruction with the patient table included in the CT, reconstruction without the patient table, and percentage difference between the two images. The color bar includes a tick for the maximum difference

**Fig. 2:**
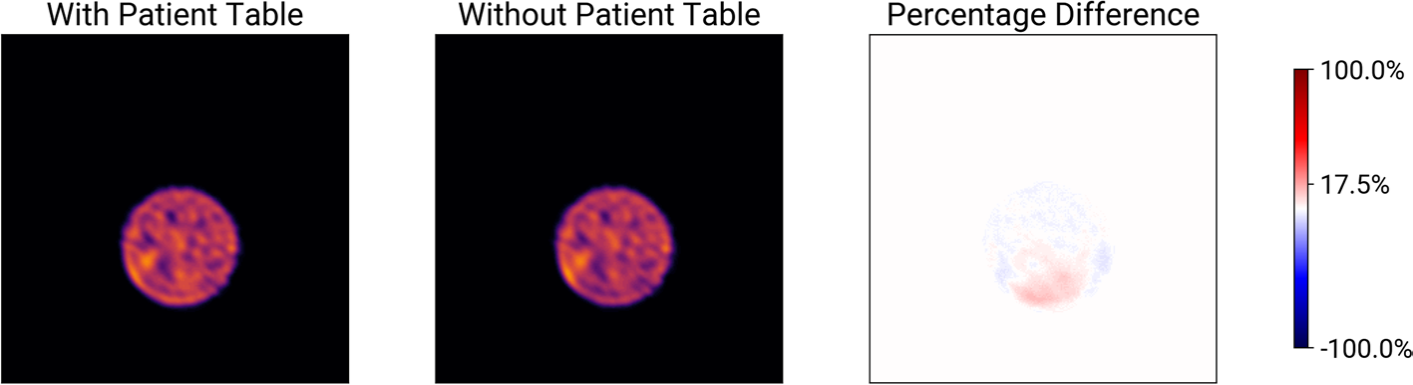
Same as Fig. 1, but reconstructions were acquired with a smartzoom collimator over a $210^\circ$ radius according to our MPI protocol

The results of the Jaszczak phantom scans with and without the patient table are shown in Fig. 1 and 2. Figure 1 shows images acquired according to our quality control protocol and Fig. 2 shows images acquired according to our MPI protocol. Both figures show the reconstruction with and without the patient table taken into account during attenuation correction, as well as the percentage difference between the two reconstructions. They show the slice with the largest percentage difference. In both cases, the reconstructions look similar. The percentage difference shows that the activity is underestimated in the lower part of the phantom when the patient table is not included in the attenuation correction. In both the quality control and the MPI procedures, the percentage difference is at most 17.5 % (see Figure 2).

**Fig. 3:**
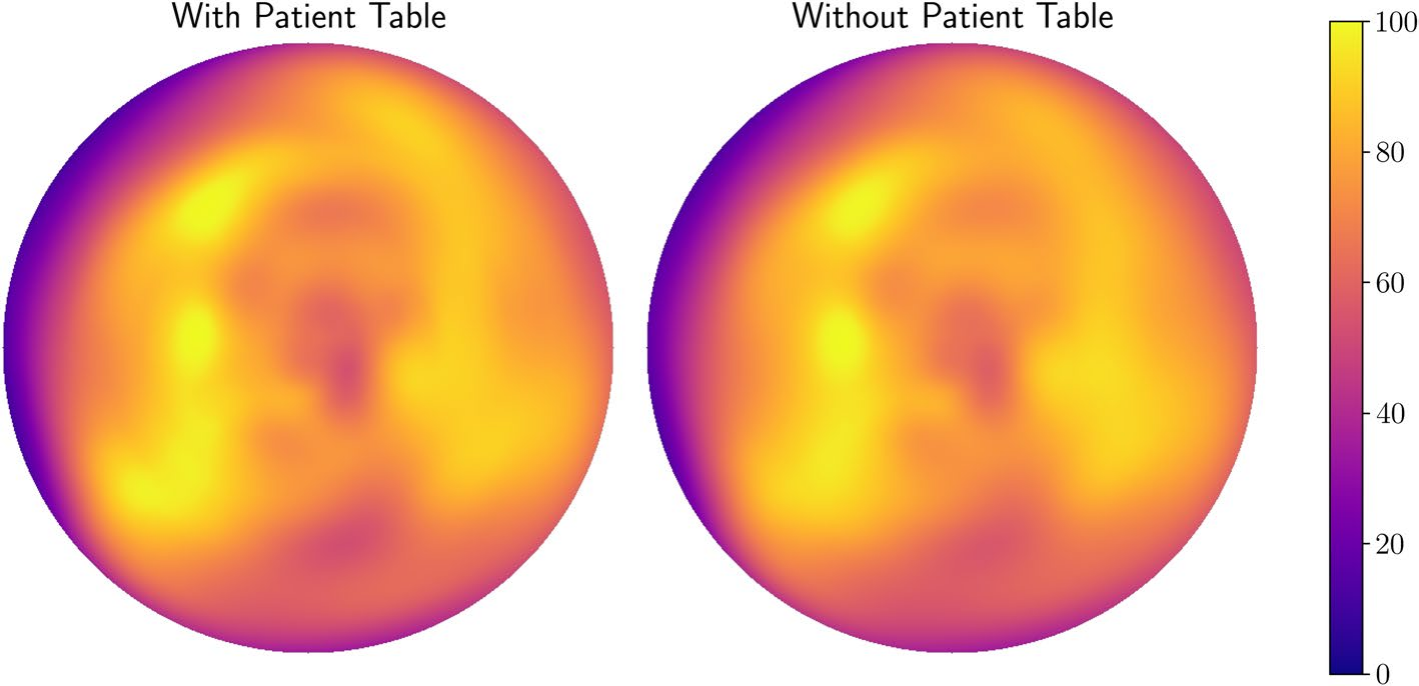
Polar maps of a stress study reconstructed with and without consideration of the patient table for attenuation correction. The patient has a small inferior ischemia.

Figure 3 shows polar maps of a patient study reconstructed with and without the patient table as part of the attenuation correction. It shows the MPI study with the largest absolute difference in segment perfusion scores in the data. Nevertheless, the polar maps look qualitatively similar.

**Fig. 4:**
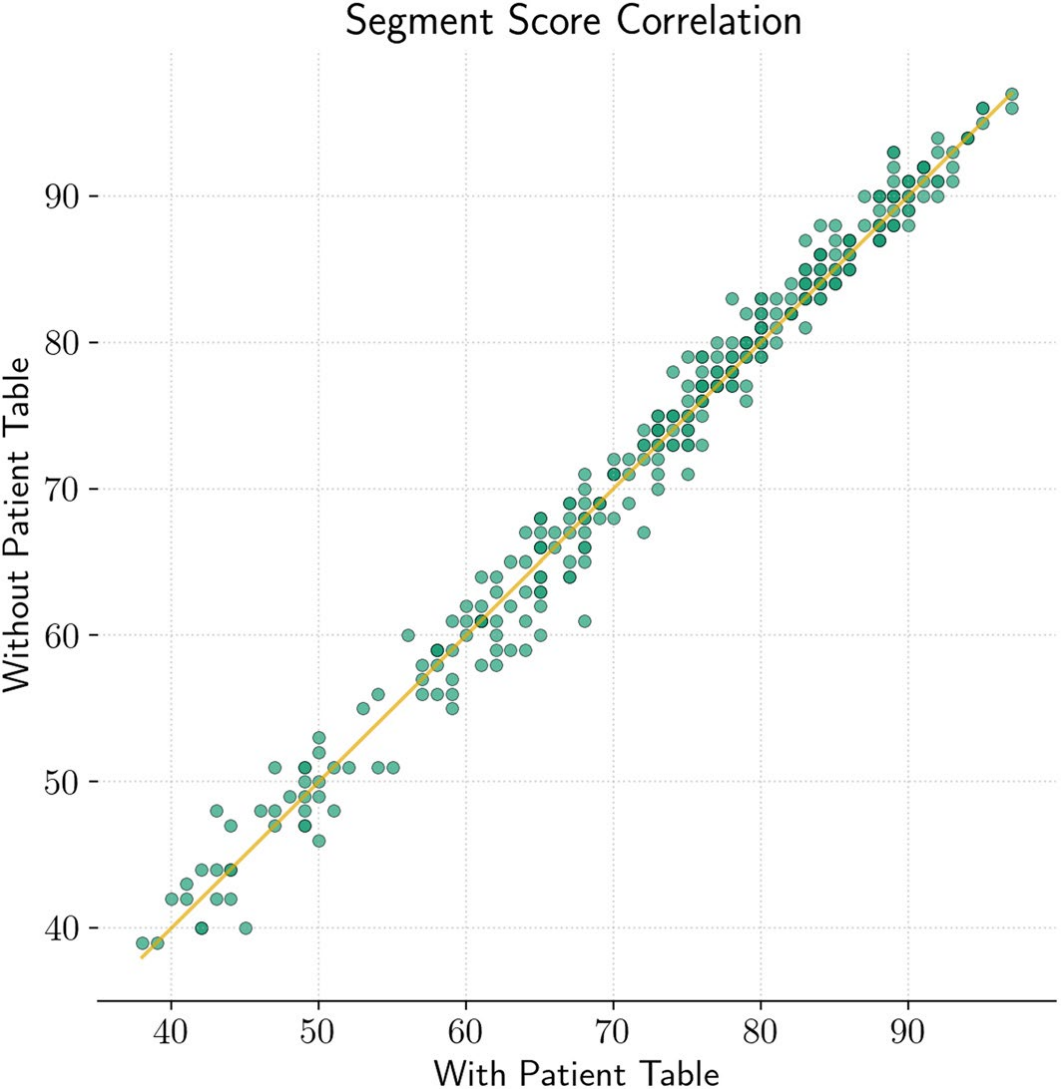
Scatter plot of segment perfusion score correlation for all studies

The scatter plot in Fig. 4 illustrates the correlation of segment perfusion scores between attenuation correction with and without the patient table. It contains one dot for each segment perfusion score of each study (19 studies with 17 segments each gives 323 dots. Each green dot indicates a combination of a segment with score X in a polar map reconstructed with attenuation correction taking the patient table into account and score Y in a polar map reconstructed with attenuation correction without taking the patient table into account. Darker colored points belong to more frequent perfusion score combinations. The yellow line is the identity line. Therefore, points below this line are cases where the segment perfusion score is lower for polar maps where the patient table was considered for attenuation correction. Scores in both conditions are normally distributed according to the Shapiro-Wilk test (both with and without patient table *p*=0.0) and there is no significant difference according to the t-test (*p*=0.83). Furthermore, the correlation coefficient is *R*=0.99.

**Fig. 5:**
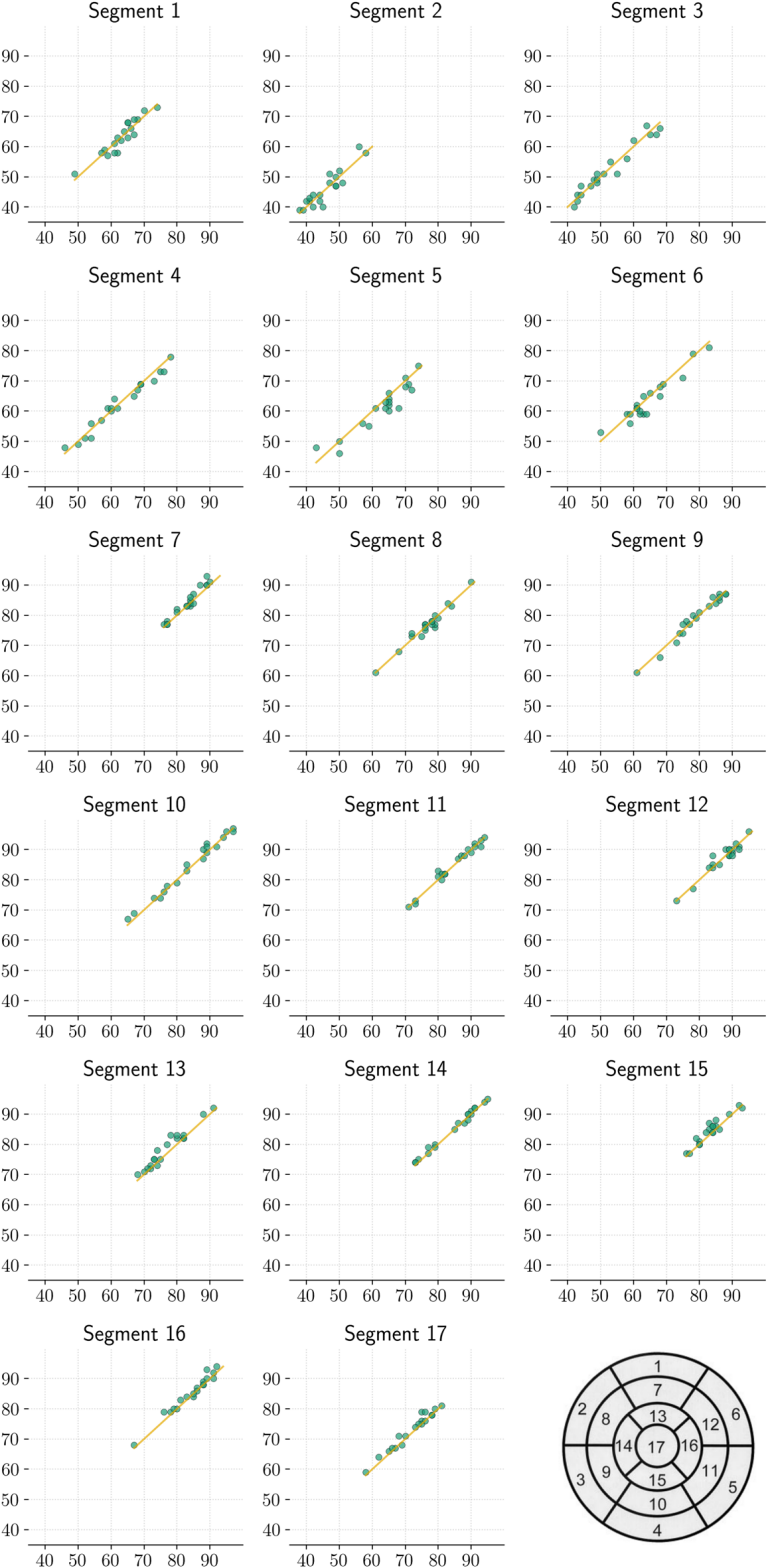
Scatter plot of segment perfusion score correlation by segment according to the AHA model. Each plot contains a dot for this segment score in each study. The location of each segment in the AHA model is depicted on the lower right

Figure 5 shows scatter plots of segment perfusion score correlation per segment according to the polar map model specified by the AHA. The position of each segment in the AHA model is depicted on the lower right. Each plot contains a dot for this segment score for each study. As in Fig. 4, the x-axis shows the segment perfusion score in the reconstruction with taking the patient table into account for attenuation correction and the y-axis without. None of the segments have a statistically significant difference. The largest differences occur in segments 5 (basal inferolateral) and 13 (apical anterior). By tendency, perfusion is overestimated in segment 5 and underestimated in segment 13 when the patient table is not taken into account for attenuation correction.

## Discussion

The results show that the effect of the patient table is small and negligible with respect to the clinical evaluation. Both the reconstructions from the phantom study and the polar maps from the patient studies look qualitatively similar. Furthermore, the differences in segment perfusion scores are small even for the segments with the largest differences. In general, there are no significant changes in segment perfusion scores when the patient table is removed from the CT for attenuation correction.

As indicated in the phantom studies, the effect of the patient table on attenuation is greatest for regions of the object (phantom or body) close to the table. The reason for this is that when looking from positions through the patient table, the effect of the phantom or body on itself is much larger for parts far away from the patient table than for parts close to the table. Therefore, only locations where the radiation passes mostly through air and the patient table, but not through the phantom or body itself, are affected. This also explains the small effect of the patient table attenuation in the patient studies. The heart is close to the front of the body, so radiation from the heart always passes through the body of the patient before it passes through the patient table, and the effect of the patient table is negligible in comparison.

An unexpected result of this study is that the effect of the patient table on attenuation is similar in the quality control and in the MPI procedures. It was expected to be higher in the quality control procedure because the acquisition of projection images over a $360^\circ$ radius means that more projection images are affected by the patient table attenuation. However, the phantom reconstructions show that the effect is similar in both protocols. This is likely due to the fact that the patient table attenuation is highest when the detector is looking sideways through the patient table [16], which is also the case for the projection images in the MPI protocol.

A limitation of this study is the small number of patients. This may also be the reason why the differences in perfusion scores in segments 5 and 13 are insignificant. However, even if the differences were significant, they would still be small. Another limitation is that although polar maps were investigated as a clinically relevant outcome, they were compared only qualitatively and by their segment perfusion scores, which is not a clinical endpoint. Nevertheless, the comparison shows that the polar maps are so similar that the consideration of a clinical endpoint may be unnecessary. A further limitation is that only data from a single scanner is considered. Therefore, this study should be replicated with data from other scanners from other vendors in other centers. However, we assume that the results can be transferred to other scanners. In general, MPI protocols do not record most projections from the side of the table where the influence of the table is greatest. Furthermore, it can be assumed that patient tables from different manufacturers have similar thicknesses and are made of similar materials, which means that they cause similar attenuation. When building a patient table for a scanner, it is natural to want to minimize its influence on the scan.

## Conclusions

This work shows that the effect of the patient table on attenuation in MPI SPECT is small. Therefore, it is not necessary for a DLAC model to predict the patient table, which may allow an easier transfer of a model from one scanner to the next. However, an important limitation of this work is that only data from a single scanner were considered. Other manufacturers may produce patient tables with different designs and materials that may have a significant effect. We do not believe that this is the case, but to be sure, this study needs to be repeated with other scanners.

## Data Availability

The datasets generated and analyzed in the current study are not publicly available, as the patients did not consent to publicly share their data. However, they are available in a pseudonymized format from the corresponding author on reasonable request.

## Footnotes

1 Available at https://gitlab.ruhr-uni-bochum.de/thuxohl_publications/2024-patient_table_attenuation/-/blob/main/data/remove_patient_table.py#L14-54
